# Evolution of different rice ecotypes and genetic basis of flooding adaptability in Deepwater rice by GWAS

**DOI:** 10.1186/s12870-022-03924-y

**Published:** 2022-11-14

**Authors:** Xueqiang Wang, Yan Zhao, Conghui Jiang, Libing Wang, Lei Chen, Fengmei Li, Yanhong Zhang, Yinghua Pan, Tianzhen Zhang

**Affiliations:** 1 Agronomy Department, College of Agriculture and Biotechnology, Zhejiang University, Hangzhou, 310058 People’s Republic of China; 2 Hainan Yazhou Bay Seed Laboratory, Sanya, Hainan 572025 People’s Republic of China; 3 Hainan Institute of Zhejiang University, Sanya, Hainan 572025 People’s Republic of China; 4 State Key Laboratory of Crop Biology, Shandong Key Laboratory of Crop Biology, College of Agronomy, Shandong Agricultural University, Tai’an, Shandong 271018 People’s Republic of China; 5 Shandong Rice Research Institute, Shandong Academy of Agricultural Sciences, Jinan, 250100 China; 6 Rice Research Institute, Guangxi Academy of Agricultural Sciences/Guangxi Key Laboratory of Rice Genetics and Breeding, Nanning, 530007 Guangxi China; 7 Institute of Nuclear and Biological Technologies, Xinjiang Academy of Agricultural Sciences, Urumqi, 830091 China

**Keywords:** Rice ecotype, Evolution, Genomic analysis, Deepwater rice, GWAS

## Abstract

**Background:**

Rice is the world’s second largest food crop and accelerated global climate change due to the intensification of human activities has a huge impact on rice. Research on the evolution of different rice ecotypes is essential for enhancing the adaptation of rice to the unpredictable environments.

**Results:**

The sequencing data of 868 cultivated and 140 wild rice accessions were used to study the domestication history and signatures of adaptation in the distinct rice ecotypes genome. The different populations had formed distinct rice ecotypes by phylogenetic analyses and were domesticated independently in the two subspecies of rice, especially deepwater and upland rice. The domestication history of distinct rice ecotypes was confirmed and the four predicted admixture events mainly involved gene flow between wild rice and cultivated rice. Importantly, we identified numerous selective sweeps that have occurred during the domestication of different rice ecotypes and one candidate gene (*LOC_Os11g21804*) for deepwater based on transcriptomic evidence. In addition, many regions of genomic differentiation between the different rice ecotypes were identified. Furthermore, the main reason for the increase in genetic diversity in the ecotypes of *xian (indica)* rice was the high proportion of alternative allele frequency in new mutations. Genome-wide association analysis revealed 28 QTLs associated with flood tolerance which contained 12 related cloned genes, and 20 candidate genes within 13 deepwater QTLs were identified by transcriptomic and haplotype analyses.

**Conclusions:**

These results enhanced our understanding of domestication history in different rice ecotypes and provided valuable insights for genetic improvement and breeding of rice in the current changing environments.

**Supplementary Information:**

The online version contains supplementary material available at 10.1186/s12870-022-03924-y.

## Background

Asian cultivated rice is one of the world’s most important food crops and features phenotypically divergent ecotypes adapted to distinct hydrological conditions [1]. Irrigated, upland, rainfed lowland and deepwater rice are the main ecotypes. In deepwater rice, internode elongation is rapid, and the leaves remain above the water surface in response to months of deep flooding; by contrast upland rice tends to have long and thick roots adapted to upland areas [2–4]. In-depth analysis of the genomes of different rice ecotypes could not only enhance our understanding of rice domestication history but also provide valuable insights that could be used to aid modern rice breeding.

Rice ecotypes have been examined using a variety of different approaches as well as from various perspectives. Drought resistance is an important trait that permits rice to adapt to most rainfed upland areas [4]. In the past few decades, many genes such as *DRO1*, *SNAC2*, *OsIAA6*, and *OsABF1* [5–9] have been reported to be related to drought resistance. The frequency of floods and the magnitude of disaster losses in countries around the world have been increasing yearly in recent decades [10]. Deepwater rice has evolved traits that permit it to survive in long periods of flooding, including its rapid internode elongation and its ability to position its leaves above the water surface, which prevents drowning [11, 12]. Many genes related to the flood tolerance of deepwater rice have been identified, such as *SD1*, *SNORKEL1*, *SNORKEL2*, and *Adh1* [3, 12–16]. These studies have generally focused on the effect of single genes, yet adaptation to dry land and deep flooding likely involves multiple genes. Thus, several studies have examined the genetic mechanisms underlying upland adaptation through genome analysis of upland and irrigated rice, and several genes related to drought resistance have been identified [17–19]. Recently, an analysis of the transcriptomic divergence between upland and lowland ecotypes suggested that many key genes involving in adaptation to drought in upland rice have the potential to be used for breeding water-saving and drought-resistant rice [20]. The authors studied genomic signatures of domestication and adaptation during geographical expansions of rice cultivation. And they found a number of rice environmental adaptability loci by GWAS using 185 wild rice (*O. rufipogon*) and 743 cultivated rice varieties, and performed transgenic verification on one of the cold-resistant loci [21]. To examine environmental factors associated with the geographic distribution of rice diversity, the authors undertook population-genomic analysis and reconstructed the ancient dispersal of rice in Asia using whole-genome sequences of more than 1400 landraces, coupled with geographic, environmental, archaeobotanical and paleoclimate data [22]. Given all these numerous papers, however, the comprehensive analyses for all these ecotypes (irrigated, upland, rainfed lowland, swamp, tidal wetland and deepwater rice, and so on) had not been done until now. Simultaneously, the domestication of some distinct cultivated types (especially deepwater rice) remains unclear. Therefore, comprehensive analyses of abundant and typical populations of rice are important for enhancing our understanding of the genetic mechanisms underlying adaptations to local environments and identifying key genes associated with different phenotypes.

Here, we studied genomic variation from two panels, 868 cultivated and 140 different types of wild rice with high sequencing depth from data published by the NCBI, to investigate the phylogenetic relationships between distinct rice ecotypes and wild rice, and identified the selection signatures in different rice ecotypes from domestication. Furthermore, the QTLs of flooding tolerance were determined by GWAS for deepwater rice, and candidate genes within the QTL were further determined by transcriptome analysis. Our analyses could provide new insights into the domestication process of different ecotypes in rice subspecies (*xian (indica)* and *geng (japonica)*). Besides, the selection signatures in different rice ecotypes and candidate genes of flooding tolerance could be used for modern rice breeding in the current changing and hostile environments.

## Results

### Geographical distribution and population structure of different cultivated rice ecotypes adapted to local water conditions

**Fig. 1:**
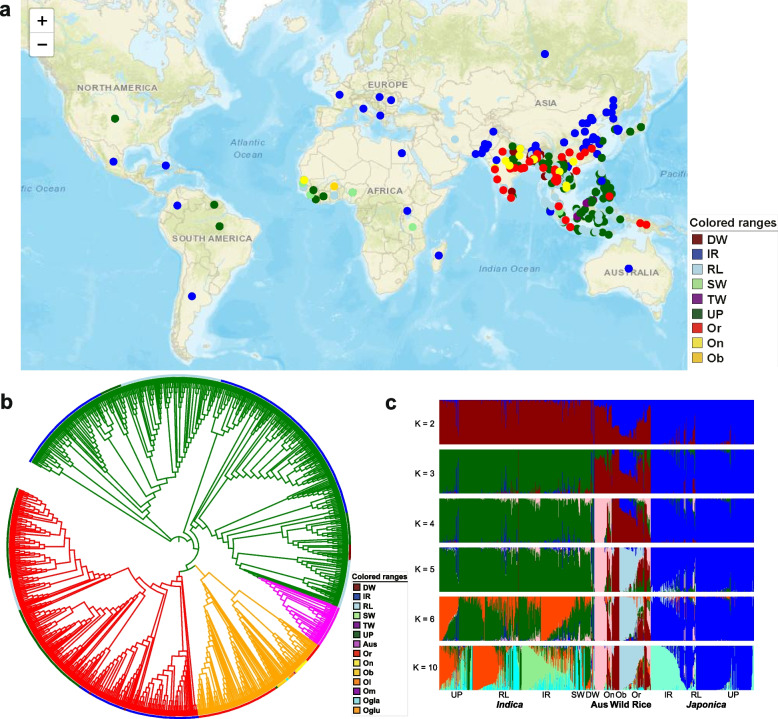
Population genomic analyses in rice. **a.** Geographic distribution of 140 wild and 868 cultivated rice accessions. Darkred, blue, lightblue, purple, lightgreen, darkgreen, red, yellow and gold dots on the world map represent the deepwater rice (DW), irrigated rice (IR), rainfed lowland (RL), tidal wetland rice (TW), swamp rice (SW), upland rice (UP), *Oryza rufipogon* (Or), *Oryza nivara* (On) and *Oryza barthii* (Ob), respectively. **b.** Phylogenetic tree of all accessions inferred from whole-genome SNPs. The major clades are indicated in the outer. The colors were same as the geographic distribution. In addition, magenta, darkorange, darkmagenta, orange and cyan represent the aus, *Oryza longistaminata* (Ol), *Oryza meridionalis* (Om), *Oryza glumaepatula* (Oglu) and *Oryza glaberrima* (Ogla). The green, red, magenta and orange lines indicate groups of XI, GJ, aus and wild rice accessions. **c.** ADMIXTURE plot for all rice accessions assuming K = 2–6 and K = 10. The values of K represent the number of clusters. At K = 10, nine subgroups of domesticated rice and three subgroups of wild rice are identified

All 867 available rice accessions with clear geographic distribution and cultivation information were collected and used to construct a rice diversity panel including six different cultivated types, 37 deepwater rice (DW), 12 swamp rice (SW), 3 tidal wetland rice (TW), 316 irrigated rice (IR), 191 rainfed lowland (RL), and 308 upland rice accessions (UP) (Fig. 1**a**, Additional file 1: Table S1). The division of cultivation types was mainly based on the local hydrological conditions, including DW under deep flooding, SW, TW and IR under fully anaerobic environment, RL under temporarily aerobic environment and UP under fully aerobic environment.

A set of high-quality SNPs from these samples, coupled with 140 wild and one cultivated rice accessions, was used to construct phylogenetic relationships; of the total SNPs, 5,707,104 (27.86%) were located at the promoter region determining the level of expression, and 493,399 (2.41%) were missense variants affecting the protein sequence (see Methods) (Additional file 2: Table S2). The topology of the phylogenetic tree presented that the *xian (indica)* and *geng (japonica)* forms of *O. sativa* were completely separated and descended from indica-type (OrI) and japonica-type wild rice groups (OrII), respectively (Fig. 1b), consistent with a previous study [23]. Among the different cultivated types, we found that within the *xian (indica)* subgroup, irrigated (181), rainfed lowland (156), tidal (3), and upland rice accessions (104) were clustered into the major *xian (indica)* branch, whereas deepwater (25) and swamp rice (12) were clustered into a single branch, respectively. There were two clear branches within the *geng (japonica)* subgroup in the Asian; one included irrigated rice (111), and another included upland (182) and rainfed lowland rice (21). Irrigated *xian (indica)* and *geng (japonica)* branches were clustered separately with their corresponding wild rice and were present across the whole planting area of *xian (indica)* rice and *geng (japonica)* rice, respectively. Swamp rice was closely related to local irrigated *xian (indica)* in Southeast Asia, whereas deepwater rice was closely related to local upland *xian (indica)* rice in South Asia. And it is worth noting that upland *geng (japonica)* rice was closely related to the irrigated *geng (japonica)*, which is broadly distributed in Asia.

Analyses of admixture and principal component (PC) based on SNP variation were performed to further characterize the genetic relationships among rice ecotypes. The *geng (japonica)*, *xian (indica)*, Aus, and wild rice were identified at *K* = 4. *Geng (japonica)*, *xian (indica)* and Aus formed one isolated cluster, whereas wild rice formed a separate, more diffuse cluster in the PC plot with the top two PCs (Additional file 3: Fig. S1). These results indicated that all *xian (indica)* ecotypes were clustered together, as well as all *geng (japonica)* ecotypes. Admixture analysis indicated that the optimal number of populations was 10. And eight rice ecotypes (upland *xian (indica)*, rainfed lowland *xian (indica)*, irrigated *xian (indica)*, swamp *xian (indica)*, deepwater *indica*, upland *geng (japonica)*, rainfed lowland *geng (japonica)* and irrigated *geng (japonica)*) and three species of wild rice were identified at *K* = 10 (Fig. 1c). All these rice ecotypes could be well distinguished in the PC plots constructed with the top three PCs. The results indicated that each rice ecotype had unique genetic characteristics. Overall, the present results suggest that deepwater *xian (indica)*, swamp *xian (indica)*, and upland *geng (japonica)* originated independently as a monophyletic group and present distinct ecotypes with unique genetic characteristics adapted to local hydrological conditions.

### Domestication processes of the distinct rice ecotypes

**Fig. 2:**
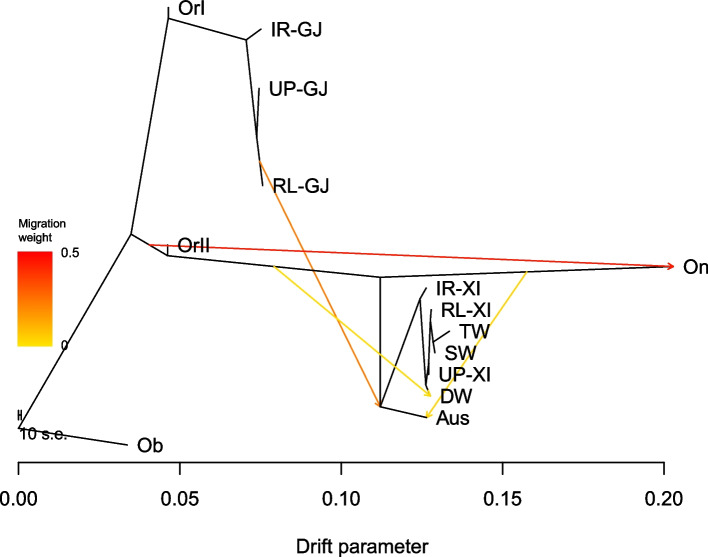
Maximum-likelihood admixture graph of rice. The wild rice population was divided into four subpopulations (*Oryza rufipogon* (OrI and OrII), *Oryza nivara* (On) and *Oryza barthii* (Ob)). The abbreviations for the major domesticated rice subgroups are deepwater rice (DW), irrigated *indica* rice (IR-XI), irrigated *japonica* rice (IR-GJ), rainfed lowland *indica* (RL-XI), rainfed lowland *japonica* (RL-GJ), tidal wetland rice (TW), swamp rice (SW), upland *indica* rice (UP-XI) and upland *japonica* rice (UP-GJ). African wild rice, *Oryza barthii* (Ob), was used to root the tree. The bootstrap values on the tree are based on 10,000 replicates. Arrows on the graph represent admixture events among different rice populations. The scale bar shows ten times the average standard error of the entries in the sample covariance matrix

The gene flow between wild and domesticated rice might distort phylogenetic relationships and affect our understanding of rice domestication [24]. To confirm the domestication processes of the distinct rice ecotypes, we used TreeMix to deeply examine the topology of relationships and migration history among different subgroups and ecotypes (Fig. 2, Additional file 3: Figs. S2-S3). Wild rice was divided into four subgroups, including *Oryza rufipogon* (OrI and OrII), *Oryza nivara* (On), and *Oryza barthii* (Ob). Domesticated rice was divided into three subgroups, *xian (indica)*, *geng (japonica)*, and Aus, among which, *geng (japonica)* was further divided into irrigated *geng (japonica)* rice (IR-GJ), rainfed lowland *geng (japonica)* rice (RL-GJ) and upland *geng (japonica)* rice (UP-GJ). *Xian (indica)* was further divided into six subgroups: deepwater *xian (indica)* rice (DW-XI), irrigated *xian (indica)* rice (IR-XI), rainfed lowland *xian (indica)* rice (RL-XI), swamp *xian (indica)* rice (SW-XI) and upland *xian (indica)* rice (UP-XI), and tidal wetland *xian (indica)* rice (TW). The number of migration events (m) allowed in the model affects the topology of the ML trees, and the subgroup graphs were constructed with the number of migration events, m = 4, given that the variance explained was greater than 99% and further increases in the number of migration events resulted in only marginal improvements in the variance explained. The ML trees showed that *geng (japonica)* was more closely related to OrI rice, whereas *xian (indica)* and Aus were more closely related to OrII and On rice (Fig. 2, Additional file 3: Figs. S2-S3). Here, the topology of the ML trees (m = 4) supported the phylogenetic relationships of rice ecotypes in the NJ tree of the 868 cultivated and 140 wild rice accessions. The four suggested admixture events mainly involved gene flows between wild rice and cultivated rice, including OrII to On, RL-G to Aus, OrII to DW, and On to Aus. These gene flows contributed 47.73, 42.85, 8.59 and 8.57% of the DNA in *Oryza nivara*, Aus, deepwater and Aus, respectively. However, no obvious admixture event was detected among rice ecotypes. These results confirmed that irrigated *xian (indica)* and irrigated *geng (japonica)* ecotypes were derived from indica-type *Oryza rufipogon* (OrI) and japonica-type *Oryza rufipogon* (OrII), and the other ecotypes of cultivated rice comprised different cultivated types adapted to different local water conditions.

### The increase in genetic diversity in Deepwater, rainfed lowland, and upland rice might be related to new mutations

To characterize whole genomic changes after the formation of deepwater rice, rainfed rice, and upland rice, we first explored the genomic differentiation between each ecotype and its immediate ancestral progenitor and genetic diversity. We found that genetic differentiation was larger between *xian (indica)* and wild rice (*F*_*ST*_ = 0.1978) than between *geng (japonica)* and wild rice (*F*_*ST*_ = 0.1872) (Additional file 4: Table S3), and the genetic diversity in *xian (indica)* and *geng (japonica)* was decreasing because only a limited number of individuals was preserved during domestication (π_XI_ = 0.0021, π_GJ_ = 0.0016 and π_or_ = 0.0045) (Additional file 5: Table S4). For each ecotype, considerable genetic differentiation was detected between deepwater *xian (indica)* and irrigated *xian (indica)* (*F*_*ST*_ = 0.081), between upland *xian (indica)* and irrigated *xian (indica)* (*F*_*ST*_ = 0.038), and between upland *geng (japonica)* and irrigated *geng (japonica)* (*F*_*ST*_ = 0.093) (Additional file 4: Table S3), indicating that deepwater *xian (indica)*, upland *xian (indica)*, and upland *geng (japonica)* represent unique ecotypes. Genetic diversity was higher in deepwater *xian (indica)* (π_DW-XI_ = 0.0024), upland *xian (indica)* (π_UP-XI_ = 0.0021), and upland *geng (japonica)* (π_UP-GJ_ = 0.0015) than in their corresponding immediate ancestral progenitors (π_IR-XI_ = 0.0019 and π_IR-GJ_ = 0.0012) (*p*_πDW-XI_πIR-XI__ < 0.0001, *p*_πUP-XI_πIR-XI__ < 0.0001 and *p*_πUP-GJ_πIR-GJ_ < 0.0001). The significant increase in genetic diversity from ancestral ecotypes to derived ecotypes suggested that no obvious bottleneck events occurred during domestication and that these ecotypes might have been subjected to balancing selection.

To explore the potential origins of genomic changes during the formation of the five new rice ecotypes (DW-XI, RL-XI, UP-XI, RL-GJ, and UP-GJ), we investigated the standing variation, new mutation, and lost variation of all the aforementioned ecotypes. Comparison of the variation and mutation between each ecotype and its immediate ancestral progenitor (see Methods) revealed that DW (3,727,609), UP-XI (4,332,898), and RL-XI (4,503,078) shared the majority of SNPs with its corresponding immediate ancestral progenitors, respectively. A high proportion of ecotype-private SNPs were detected in deepwater (637,570), rainfed *xian (indica)* (1,543,225), upland *xian (indica)* (1,034,495), rainfed *geng (japonica)* (457,711), and upland *geng (japonica)* (1,444,332). Analysis of alternative allele frequency differentiation (AFD) between each ecotype and its immediate ancestral progenitor was performed on standing variation, new mutations and lost variation. Importantly, the proportion of new mutations in DW derived from its immediate ancestral progenitor (0.1763) (IR-XI) was higher compared with UP-XI (0.1055) and RL-XI (0.0863); the proportion of new mutations in RL-GJ derived from its immediate ancestral progenitor (0.1349) (IR-GJ) was higher compared with UP-GJ (0.0417). AFD in standing variation between ecotypes in *geng (japonica)* (RL-GJ, UP-GJ) and its immediate ancestral progenitor (0.2618, 0.1729) was much higher compared with ecotypes in *xian (indica)* (DW, RL-XI, UP- XI) and its immediate ancestral progenitor (0.0482, 0.0265, 0.0252) (Additional file 6: Table S5).

We speculated that standing variation and new mutations may contribute to the evolution of the unique ecotypes. The main reason for the increase in the genetic diversity in the ecotypes of *xian (indica)* rice was the high proportion of alternative alleles among the new mutations relative to the low proportion of standing variation. Compared with *xian (indica)* rice, both types of variation (new mutations and standing variation) may affect the formation of *geng (japonica)* rice ecotypes. The evolution of unique *xian (indica)* and *geng (japonica)* rice ecotypes adapted to different environments might be explained by the fact that they have experienced different forms of selection.

### Signatures of selection in the genomes of different rice ecotypes

**Fig. 3:**
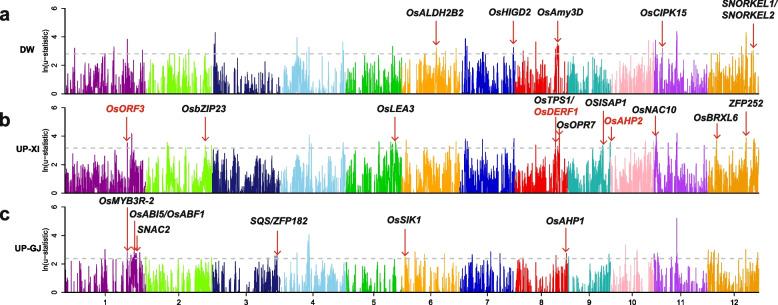
Genome-wide detection of positive selection in different rice ecotypes. **a.** deepwater rice (DW) **b.** upland XI rice **c** upland GJ rice. Dashed lines indicate the top 1% of the μ-statistic values, and red vertical bars indicate known genes involving in the flooding or drought tolerance mentioned in the text. Red arrows indicate known genes involving in the flooding or drought tolerance mentioned in the text and red gene names in **b** represent common with **c**. Red vertical bars indicate 100-kb selective sweep region on chromosome 11 suffered parallel selection during domestication of both rice ecotypes

To detect positive selection for distinctive rice ecotypes (DW, UP-XI, UP-GJ, IR-XI and IR-GJ), we defined the region scoring in the top 1% of the μ-statistic values as candidate positive selective sweeps using RAiSD [25]. We merged adjacent regions that exceeded the threshold into a single region, and identified 52, 57, and 61 regions subject to positive selection in DW, UP-XI, and UP-GJ, respectively (Fig. 3, Additional file 3: Fig. S4, Additional files 7-9: Tables S6-S8). The regions subjected to positive selection in DW contained six cloned genes (*OsALDH2B2*, *OsHIGD2*, *OsAmy3D*, *OsCIPK15*, *SNORKEL1*, and *SNORKEL2*) with functions related to flood resistance, and 19 cloned genes with functions related to rice growth and development, root system, domestication, and resistance (Fig. 3a, Additional file 7: Table S6). For example, *sh4* is a major gene affecting rice shattering [26]. The regions that have experienced positive selection in UP-XI and UP-GJ contained 12 and 11 cloned genes with functions related to drought resistance, and 32 and 38 cloned genes with functions related to rice growth and development, root system, domestication, resistance, photoperiod, and meiosis (Fig. 3b and c, Additional files 8-9: Tables S7-S8). Subsequent work on these candidate genes was needed to determine whether these genes affect the domestication of different rice ecotypes (DW, UP-XI, and UP-GJ) and agronomically important traits.

**Fig. 4:**
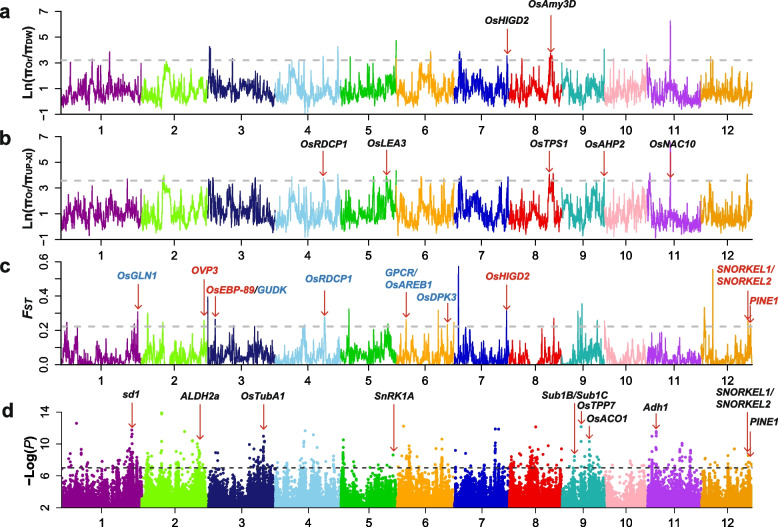
Selective sweep of different rice ecotypes and GWAS analyses on deepwater rice. **a**, **b** Selective sweep regions identified by the greatest reduction of diversity (ROD) of DW and UP-XI, respectively. **c.** Genomic differentiation regions identified by the greatest relative divergence (*F*_*ST*_) between DW and UP-XI populations. Dashed lines indicate the top 1% of the ROD or *F*_*ST*_ values, and red lines indicate known genes involving in the flooding or drought tolerance mentioned in the text. Red and blue gene names represent detected in DW and UP-XI. **d.** Manhattan plots obtained from GWAS for the deepwater rice. Dashed lines indicated the threshold for GWAS (−log(*P*) = 7). Red lines show known genes involving in the flooding mentioned in the text

To detect selective sweeps in the different rice ecotypes (DW, UP-XI, and UP-GJ), the reduction of diversity (ROD) of π_Or_/π_DW_, π_Or_/π_UP-XI_, and π_Or_/π_UP-GJ_ was calculated. The regions that were in the top 1% of the ROD were defined as candidate domestication sweeps. A total of 26, 30, and 49 potential selective sweeps of DW, UP-XI, and UP-GJ in the top 1% of the ROD were detected, which accounted for 1.42, 1.71, and 2.06% (5.30 Mb, 6.40 Mb, and 7.70 Mb) of the genome, respectively (Fig. 4a and b, Additional file 3: Fig. S5, Additional files 10-12: Tables S9-S11). The potential selective sweep regions of DW contained two cloned genes with functions related to flood resistance. Among them, *OsHIGD2* (*Oryza sativa* hypoxia-induced gene domain 1) was involved in the early signaling of hypoxia-promoted stem growth in deepwater rice, and the expression of *OsAmy3D* was enhanced during rice embryo germination under hypoxia (Fig. 4a, Additional file 10: Table S9). By contrast, the potential selective sweep regions of UP-XI and UP-GJ contained more cloned genes (5 and 12) with functions related to drought resistance, including *OsNAC10*, *OsbZIP16*, *ZFP185*, *ZFP182*, and *SQS* (Fig. 4b, Additional file 3: Fig. S5, Additional files 11-12: Tables S10-S11). KEGG pathway enrichment analysis showed that 727 genes underlying the potential selective sweeps regions of DW involved in metabolic pathways, biosynthesis of secondary metabolites, nitrogen metabolism, and phenylpropanoid biosynthesis (Additional file 13: Table S12). Whereas, the potential selective sweep regions of UP-XI and UP-GJ contained 1039 and 1147 genes, respectively, with functions related to metabolic pathways, biosynthesis of secondary metabolites, ribosome, biosynthesis of cofactors, spliceosome, carbon metabolism, and RNA transport (Additional files 14-15: Tables S13-S14). These results indicated that adaptation to the local environment in the different rice ecotypes (DW, UP-XI, and UP-GJ) has been mediated by natural and artificial selections.

Some of the same regions have experienced selection in several rice ecotypes, including DW, UP-XI, UP-GJ, IR-XI, and IR-GJ (Fig. 4a and b, Additional file 3: Figs. S5-S7, Additional files 16-17: Tables S15-S16). An about 200-kb selective sweep region on chromosome 11 was the most common, which contained 5 genes that have not been previously cloned. Among them, LOC_Os11g21804 encoding an expressed protein, and the other four genes encoding retrotransposon proteins. To further identify candidate gene within this region for deepwater rice, transcriptome analysis for deepwater and non-deepwater rice accessions was performed. The LOC_Os11g21804 was a down-regulated gene, since its RPKM_Deepwater_/RPKM_Non-deepwater_ was only 0.36 between the deepwater, and non-deepwater rice accessions subjected to complete submergence in our transcriptomic analysis, and the ratio of RPKM in the after to before submergence of the deepwater variety (RPKM_after/before_) was 0.62 (Additional file 18: Table S17). The RPKM_Deepwater_ and RPKM_Non-deepwater_ of other four genes were both zero. Moreover, the RPKM in the after and before submergence of the deepwater variety were also both zero. Therefore, this gene appeared to be the most likely candidate gene for deepwater based on the transcriptomic evidence. Moreover, this region might have experienced parallel selection during the domestication of both rice ecotypes, and these genes have played a key role in the evolution of each rice ecotype and need further in-depth analysis.

As the mean *F*_*ST*_ value between DW and UP-XI was 0.10, we searched for regions of the genome that differed between DW and UP-XI. *F*_*ST*_ throughout the genome was determined to identify divergent regions using 100-kb sliding windows and plotted using R scripts. A total of 32 divergent regions were detected, and six cloned genes with functions related to flood resistance and six cloned genes with functions related to drought resistance were located in these regions (Fig. 4c, Additional file 19: Table S18). Meanwhile, a total of 33 and 24 divergent regions were detected between IR-XI and UP-XI and between IR-GJ and UP-GJ, and these regions contained seven and ten cloned genes related to drought resistance, respectively (Additional file 3: Figs. S8-S9, Additional file 20-21: Tables S19-S20). These results indicated that these divergent regions played an important role in the adaptation of different rice ecotypes (DW, UP-XI, and UP-GJ) to the local environment.

### GWAS of the Deepwater ecotype rice

The deepwater rice has formed a distinct rice ecotype as there was high genetic differentiation between deepwater rice and other types of cultivated rice according to the phylogenetic, population structure and introgression analysis. And the deepwater rice was an extreme type of rice ecotype, so we assumed that the phenotype of deepwater rice (DW) was set to 1; all others were set to 0. We performed a mixed-model approach for the phenotype using the factored spectrally transformed linear mixed models (FaST-LMM) program with 2,282,266 SNPs after they were filtered using the following criteria: missing rate ≥ 50% and minor allele frequencies ≤5%. The first three PCs were used as covariates within the GWAS model to control the subpopulation structure (see Methods). The suggestive significant threshold was set at -log (*P*) = 7 to detect significant association signals. A total of 28 signals significantly associated with the deepwater trait distributed were detected on eleven chromosomes (except chromosome 10), and 12 cloned genes related to deep water adaptation were among the QTLs detected in our GWAS (Fig. 4d, Additional file 3: Fig. S10). Among them, *Adh1* encoding alcohol dehydrogenase promoted coleoptile elongation under submergence in rice [16]; *ALDH2a* (rice aldehyde dehydrogenase) is induced by flooding stress, and its expression is up-regulated in young leaves upon flooding stress and ABA treatment [27]. *PINE1*(*DEC1*) encoding a zinc finger transcription factor; up-regulation of its expression inhibited internode elongation, and down-regulation of its expression promoted internode elongation [28, 29]. *Sub1B* and *Sub1C* are ethylene-responsive transcriptional factors [28–30]; *SNORKEL1* and *SNORKEL2* are ethylene response factors that could promote the adaptation of rice to deep water environments (Additional file 22: Table S21) [13, 28]. Interestingly, *sd1* (a gibberellin biosynthesis gene) controls plant height as well as submergence-induced internode elongation [3]. Therefore, we believed that the approach of GWAS was feasible and the results were credible.

**Fig. 5:**
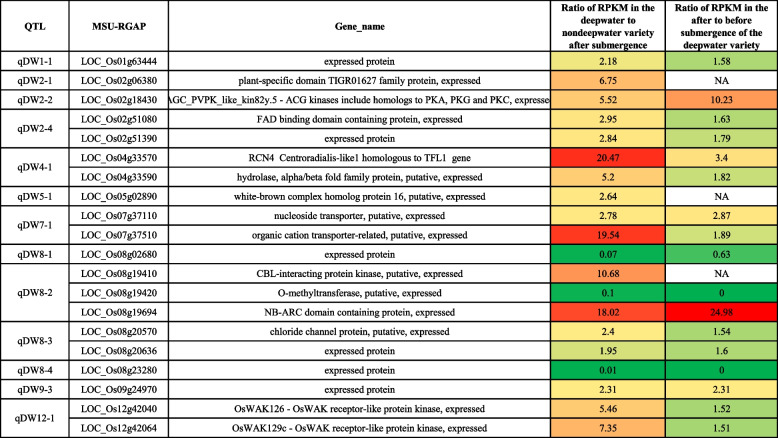
The candidate genes for deepwater QTLs and heat map of the ratio of RPKM. Different colors show ratio of RPKM in the deepwater to nondeepwater varieties subjected to complete submergence, and the ratios of RPKM of these genes in the after and before submergence of the deepwater variety (RPKM_after/before_). Rows of the heat map correspond to the 20 candidate genes for deepwater QTLs listed on the left of the table

To further determine the function of these genes, we compared RPKM_Deepwater_ to RPKM_Non-deepwater_ between the deepwater and non-deepwater rice accessions subjected to complete submergence in our transcriptomic analysis. The genes detected in GWAS for deepwater rice were up-regulated or down-regulated genes, since their RPKM_Deepwater_/RPKM_Non-deepwater_ were greater than 1.5 or lower than 0.67 between the deepwater and non-deepwater rice accessions subjected to complete submergence (Fig. 5). Moreover, the ratios of RPKM of these genes in the after and before submergence of the deepwater variety (RPKM_after/before_) were consistent with those described above. Among them, eight genes (*sd1*, *ALDH2a*, *OsTubA1*, *Sub1B*, *Sub1C*, *OsTPP7*, *OsACO1* and *Adh1*) were up-regulated genes, while only two genes (*SnRK1A* and *PINE1*) were down-regulated genes (Fig. 5, Additional file 23: Table S22). Meanwhile, the comparison for RPKM of other cloned genes for flooding tolerance was performed as described above. The RPKM_Deepwater_/RPKM_Non-deepwater_ and RPKM_after/before_ in the deepwater variety of all other cloned genes for flooding tolerance were greater than 1.5 or lower than 0.67 between the deepwater and non-deepwater rice accessions (Fig. 5, Additional file 23: Table S22). Such as *ACE1* (ACCELERATOR OF INTERNODE ELONGATION 1) controls stem elongation in response to gibberellin production. So, we believed that the results of transcriptomic analyses were credible and could be used to further identify candidate genes for QTLs detected by GWAS.

Since we have proved that the results of transcriptomic analyses were credible as described above. To further identify candidate genes within the QTL for deepwater rice, the comparison for the RPKM of genes within each QTL detected by GWAS between the deepwater and non-deepwater rice accessions, and the ratios of RPKM of these genes in the after and before submergence of the deepwater variety (RPKM_after/before_) were analyzed. Based on the transcriptomic data, we identified 20 candidate genes for 13 deepwater QTLs detected by GWAS. Among them, 17 genes were up-regulated genes, while 3 genes were down-regulated genes (Fig. 5). And haplotype analysis was performed for all genes as above. The haplotypes of these genes in deepwater rice were distinctly different from those in other ecotypes and wild rice (Additional file 24: Table S23). Therefore, these genes appeared to be the most likely candidate gene for deepwater based on these evidences. In addition, these genes could be used in future studies for examining the genetic mechanism underlying deepwater traits.

## Discussion

### Domestication models of rice

In this paper, we showed that there are several ecotypes of cultivated rice adapted to different local water conditions. This work provided new insights into the domestication process of different rice ecotypes using a large amount of genomic data from wild and cultivated rice. Several domestication selective sweeps and putative causal genes for rice ecotypes and some divergent regions between rice ecotypes were detected, and this new information will greatly aid modern rice breeding. The multiple origin model of Asian rice based on phylogenetic analyses of different rice subpopulations using different sets of markers in different rice collections predicted that *xian (indica)*, *geng (japonica)*, and Aus are more closely related to distinct *O. rufipogon* or *O. nivara* populations rather than to each other [23, 24, 31–36].

The results of the genome-wide analysis of 868 cultivated and 140 wild rice accessions are consistent with the results of the phylogenetic analysis and PCA, all of which support multiple origins for different rice subpopulations. Phylogenetic analysis and PCA revealed that the different rice ecotypes in *xian (indica)* and *geng (japonica)* formed a cluster that was more closely related to OrI and OrII/On, respectively, and Aus was more closely related to OrII/On. These findings were consistent with the results that the *xian (indica)*, *geng (japonica)*, and Aus have been independently domesticated [35]. Moreover, the TreeMix results indicated a dual origin of domesticated *xian (indica)* and *geng (japonica)*; the one inconsistency with previous studies was that different ecotypes in *xian (indica)* rice and Aus were derived from crosses between OrII and On wild rice [23].

### Genetic basis of rice flooding adaptability and selection signals of different rice ecotypes

So far, 26 genes for flooding stress response have been cloned by forward or reverse genetic strategies in rice [3, 12–16]. The resequencing provides vast natural variations, and the candidate genes in natural populations could be explored by GWAS [3, 37, 38]. The 12 previously known cloned genes related to deep water adaptation were detected in our GWAS. However, the previously flooding stress response genes identified using mutants and gene differential expression with no mutations or few variations in our populations were not detected. Therefore, different populations may be required to detect different functional genes using population genetic, transcriptome analysis and transgenic validation, and these genes could be used for examining the genetic mechanism underlying deepwater traits.

The potential selective sweeps and positive selective sweeps detected of DW contained two common cloned genes (*OsHIGD2* and *OsAmy3D*) with functions related to flood resistance; the other four genes (*OsALDH2B2*, *OsCIPK15*, *SNORKEL1*, and *SNORKEL2*) with functions related to flood resistance were only detected in positive selective sweeps. Similarly, the potential selective sweeps and positive selective sweeps detected in UP-GJ consisted of three cloned genes (*SQS*, *ZFP182*, and *OsAHP2*) with functions related to drought resistance; eight genes (*OsERF3*, *OsMYB3R-2*, *OsABI5*, *OsABF1*, *SNAC2*, *OsSIK1*, *OsDERF1*, and *OsAHP1*) were only detected in positive selective sweeps, and a different set of eight genes (*OsbZIP16*, *ZFP185*, *OsSKIPa*, *OsDREB2B*, *OsbZIP52*, *OsOPR7*, *RSOsPR10*, and *OsPTR2*) were only detected in selective sweeps. By contrast, a total of 29, 48, 18 and 37 selective sweep regions detected in UP-XI, UP-GJ, IR-XI and IR-GJ, respectively, were inconsistent with the results in previous studies [17, 19]. The results of two methods for identifying selective sweeps for DW and UP-GJ and the divergent regions between DW and UP-XI using different sample sets were inconsistent. Therefore, a comprehensive analysis using different collections and methods is needed to enhance our understanding of the genetic mechanisms underlying the evolution of different rice ecotypes.

### Parallel selection on specific regions of the genome during the domestication of different rice ecotypes

Parallel evolution is a widespread and well-studied phenomenon in plants [39–41]. The domestication of upland rice might have involved the evolution of different adaptive strategies in response to different artificial selection pressures in *xian (indica)* and *geng (japonica)*, as suggested by a previous study that explored the parallelism of genetic changes during upland rice domestication [42]. Although many studies have been conducted, the patterns of selection that have fostered the evolution of different rice ecotypes during domestication remain unclear. The systematic analysis of all different ecotypes has not been conducted and the domestication of some distinct cultivated types (especially deepwater rice) remains unclear. In this study, we found that some of the same regions were under selection in several rice ecotypes, including DW, UP-XI, UP-GJ, IR-XI, and IR-GJ. And *LOC_Os11g21804* appeared to be the most likely candidate gene for deepwater based on the transcriptomic evidence. Therefore, these regions might have experienced parallel selection during the domestication of rice ecotypes, and this played a key role in the evolution of each rice ecotype. Balancing selection was detected in deepwater rice. These selection signatures in different rice ecotypes provide valuable insights that could be used to aid modern rice breeding in the current changing environments. Furthermore, a comprehensive genomic analysis combined with deepwater and salt tolerance can also provide a very valuable theoretical reference for saline-alkali tolerant rice breeding.

## Conclusion

In conclusion, we showed the different populations had formed distinct rice ecotypes and were domesticated independently in the two subspecies of rice, especially deepwater and upland rice. The domestication history of distinct rice ecotypes was confirmed and the four predicted admixture events mainly involved gene flow between wild rice and cultivated rice. In addition, the main reason for the increase in genetic diversity in the ecotypes of *xian (indica)* rice (XI) was the high proportion of alternative allele frequency in new mutations relative to the low level of AFD between each ecotype and its immediate ancestral progenitor in standing variation. Importantly, we identified numerous selective sweeps that have occurred during the domestication of different rice ecotypes. Especially, a 200-kb selective sweep region on chromosome 11 suffered from parallel selection during domestication of both rice ecotypes, which contained 5 genes that have not been previously cloned and thus need further in-depth analysis. Moreover, *LOC_Os11g21804* appeared to be the most likely candidate gene for deepwater based on the transcriptomic evidence. Genome-wide association analysis revealed 28 quantitative trait loci associated with flood tolerance which contained 12 related cloned genes. And we identified 20 candidate genes within 13 deepwater QTLs by transcriptomic and haplotype analyses.

## Methods

### Materials and sequencing data

The information on the ecotype for these rice accessions was obtained from the Catalog of Rice Germplasm Resources in China and IRRI. The whole genome sequence data of the 867 accessions were obtained from the 3000 Rice Genome (3 K-RG), which had an average sequencing depth of 14 × and mapping coverage of 94% when aligned to the Nipponbare reference genome [43–45]. A total of 15,963,233 SNPs of 867 rice genotypes were obtained from the 3000 Rice Genome (3 K-RG).

To analyze the population structure and evolutionary history of rice domestication, we obtained additional data from a publicly available set of wild rice accessions from the National Center for Biotechnology Information (NCBI). A total of one cultivated and 140 wild rice accessions with average sequencing depth greater than 12 × were used in this study, including 101 *Oryza rufipogon* (Or), 16 *Oryza nivara* (On), 15 *Oryza barthii* (Ob), 6 *Oryza longistaminata* (Ol), and one for each of the *Oryza meridionalis* (Om), *Oryza glumaepatula* (Oglu), and one African cultivated rice (*Oryza glaberrima* (Ogla)). The variants (SNPs and indels) were obtained using Trimmomatic [46], BWA [24, 47], SAMtools [48] and bcftools [49, 50] with default settings. Finally, we obtained a total of 29,965,092 SNPs from the 140 wild and cultivated rice accessions.

### Phylogenetic and population structure analysis

Sequencing data of 868 cultivated and 140 wild rice accessions were merged and further screened by in-house Perl and Python scripts. To infer the basal group of rice, a total of 3,267,963 SNPs with a missing rate of ≤50% were used to construct a phylogenetic tree by the unweighted neighbor-joining (NJ) method using the R package ‘phangorn’. Visualization and annotation for the NJ tree were conducted on the iTOL website [51].

For population structure analysis of 868 cultivated and 140 wild rice accessions, a total of 3,267,963 SNPs with a missing rate of ≤50% were screened as described above. The program GAPIT with default settings was used to infer the principal components of the 868 cultivated and 140 wild rice accessions [52]. We carried out PCA using R (version 4.0.3) and the first three eigenvectors were plotted. To analyze the population structure of these rice accessions, we performed maximum likelihood clustering analysis using ADMIXTURE (version 1.3) [53] with the 109,917 independent SNPs using a linkage disequilibrium pruning procedure with PLINK with the parameter ‘--indep-pairwise 50 5 0.3’ [54], and the genetic ancestry of each sample was estimated by the varying levels of K (K = 2–20) and running the cross-validation error (CV) procedure. K = 10 was determined to be optimal according to the cross-validation error. The result from ADMIXTURE was plotted using an R script.

### Population genetics analysis and selection signatures detection

Based on phylogenetic analysis and population structure, we defined three groups of individuals: wild rice (Ob, Or and On), *xian (indica)* rice (DW, IR-XI, RL-XI, UP-XI, and SW) and *geng (japonica)* rice (IR-GJ, RL-GJ, and UP-GJ). A total of 3,267,963 SNPs with a missing rate of ≤50% were used as described above. Nucleotide diversity (θπ) was determined for twelve populations using VCFtools [49] with a 100-kb sliding window and a 50-kb step size. Genetic differentiation (*F*_*ST*_) was calculated among different populations using the same method. To detect selective sweeps, the reduction of diversity (ROD) and *F*_*ST*_ value within the same sliding windows were calculated and we defined the regions that scored in the top 1% of the ROD and *F*_*ST*_ values as candidate domestication sweeps. Positive selection was detected based on multiple signatures of a selective sweep via the enumeration of SNP vectors in RAiSD (Raised Accuracy in Sweep Detection) [25], an open-source software that implements a novel and parameter-free detection mechanism relies on multiple signatures of a selective sweep, by non-overlapping 50-kb windows with the parameter set as ‘-M 3 -y 2 -G -A 0.995 -f’. We defined the regions that scored in the top 1% of the μ-statistic values as candidate positive selective sweeps.

### Introgression analyses for wild and cultivated rice

To further our understanding of population introgression in rice and build population trees in the presence of admixture, a subgroup graph was constructed using TreeMix [55] that allows 0–10 migration events (m), and the maximum likelihood (ML) method based on a Gaussian model of allele frequency change was used. The model with the optimal number of admixture events, m = 4, was used based on the lower corresponding residual. The subgroup graphs were constructed with the number of migration events, m = 4, which were consistent with the NJ tree of the 868 cultivated and 140 wild rice accessions as described above.

### Variation types between each ecotype and its immediate ancestral progenitor

Based on the phylogenetic analysis and population structure, 561 typical cultivated rice with the Q value greater than 0.8 using ADMIXTURE and 140 wild rice accessions were used to characterize variation types between each ecotype and its immediate ancestral progenitor. A total of 3,267,963 SNPs was generated for follow-up analysis by the union between the SNP sets of 561 cultivated rice and 140 wild rice accessions. The common SNP sets between each ecotype and its immediate ancestral progenitor were defined as standing variation, the SNP sets in only one ecotype and lacking in its immediate ancestral progenitor were defined as new mutations, and the SNP sets only in its immediate ancestral progenitor and no variation in one ecotype were defined as lost variation. The alternative allele frequency in each ecotype was calculated as the ratio of the number of alternative alleles to reference alleles.

### Genome-wide association study

Based on the phylogenetic analysis and population structure, 561 typical cultivated rice with the Q value greater than 0.8 using ADMIXTURE were used to construct the association panel. A total of 2,317,387 SNPs were obtained with a missing rate of ≤50% and minor allele frequencies of ≥5%. Moreover, the deepwater rice has formed a distinct rice ecotype as there was high genetic differentiation between deepwater rice and other types of cultivated rice according to the phylogenetic, population structure and introgression analysis. And the deepwater rice was an extreme type of rice ecotype, so we assumed that the phenotype of deepwater rice (DW) was set to 1; all others were set to 0. We used a mixed-model approach for the phenotype using the factored spectrally transformed linear mixed models (FaST-LMM) program [56]. Independent SNPs were further called using Plink v1.90b4 with the parameter ‘--indep-pairwise 50 5 0.3’, and the effective number of independent SNPs was 109,917. Suggestive threshold was calculated using the formula “-log10(0.01 / effective number of independent SNPs)” as described previously [37, 57]. Thus, the threshold -log (*P*) = 7 was used to detect significant signals for deepwater rice.

### Transcriptome analysis

To further identify candidate genes within the QTL for deepwater rice, transcriptome analysis for deepwater and non-deepwater rice accessions was performed. The transcriptome sequencing data of the deepwater rice variety (C9285) and the non-deepwater rice variety (T65) subjected to complete submergence were downloaded from the public database (PRJDB5294). And the other transcriptome sequencing data of the deepwater rice variety (8391) subjected to complete submergence (the entire aboveground shoot of 7-day-old seedlings) and under air condition were downloaded from the public database (PRJEB10405). Mapping of RNAseq reads and transcript abundance RPKM was performed by the Cufflinks. The thresholds of (RPKM, Reads Per Kilobase per Million mapped reads) RPKM_Deepwater_/RPKM_Non-deepwater_ between the deepwater (C9285) and non-deepwater rice accessions (T65) subjected to complete submergence, and the ratio of RPKM in the after and before submergence of the deepwater variety (8391) (RPKM_after/before_) > 1.5 or < 0.67 in our transcriptomic analysis was determined [38]. The up-regulated gene was defined as RPKM_Deepwater_/RPKM_Non-deepwater_ > 1.5, and down-regulated gene was defined as RPKM_Deepwater_/RPKM_Non-deepwater_ < 0.67.

## Supplementary Information

**Additional file 1 Table S1.** Information about samples used in this study.

**Additional file 2 Table S2.** Summary of Annotation for genomic variation.

**Additional file 3 Fig. S1** PCA plot of the first two eigenvectors (**a**) and two and three eigenvectors (**b**) of rice accessions. **Additional file 2. Fig. S2** Maximum-likelihood tree of rice assuming 0 to 3 migration edges. **Additional file 2. Fig. S3** Corresponding residual of rice assuming 0 to 10 migration edges. **Additional file 2. Fig. S4** Genome-wide detection of positive selection in different rice ecotypes. **Additional file 2. Fig. S5** Selective sweep regions identified by the greatest reduction of diversity (ROD) of UP-GJ. **Additional file 2. Fig. S6** Selective sweep regions identified by the greatest reduction of diversity (ROD) of IR-XI. **Additional file 2. Fig. S7** Selective sweep regions identified by the greatest reduction of diversity (ROD) of IR-GJ. **Additional file 2. Fig. S8** Genomic differentiation regions identified by the greatest relative divergence (FST) between IR-XI and UP-XI populations. **Additional file 2. Fig. S9** Genomic differentiation regions identified by the greatest relative divergence (FST) between IR-GJ and UP-GJ populations. **Additional file 2. Fig. S10** Quantile-quantile plots and Manhattan plots for the GWAS in the full populations using FaST-LMM.

**Additional file 4 Table S3.** Genetic divergence (*F*_*ST*_) among distinct rice ecotypes populations and wild rice.

**Additional file 5 Table S4.** The mean (θπ) of different subpopulations in cultivated and wild rice.

**Additional file 6 Table S5.** Variation types between each ecotype and its immediate ancestral progenitor.

**Additional file 7 Table S6.** Genome-wide detection of positive selection in deepwater (DW).

**Additional file 8 Table S7.** Genome-wide detection of positive selection in upland XI rice.

**Additional file 9 Table S8.** Genome-wide detection of positive selection in upland GJ rice.

**Additional file 10 Table S9.** Genome-wide detection and functional annotation of selective sweep regions in the deepwater rice (DW).

**Additional file 11 Table S10.** Genome-wide detection and functional annotation of selective sweep regions in the upland XI rice.

**Additional file 12 Table S11.** Genome-wide detection and functional annotation of selective sweep regions in the upland GJ rice.

**Additional file 13 Table S12.** KEGG annotation of selective sweep regions in the deepwater rice (DW).

**Additional file 14 Table S13.** KEGG annotation of selective sweep regions in the upland XI rice.

**Additional file 15 Table S14.** KEGG annotation of selective sweep regions in the upland GJ rice.

**Additional file 16 Table S15.** Genome-wide detection and functional annotation of selective sweep regions in the irrigated XI rice.

**Additional file 17 Table S16.** Genome-wide detection and functional annotation of selective sweep regions in the irrigated GJ rice.

**Additional file 18 Table S17.** Ratio of RPKM within the 200-kb selective sweep region on chromosome 11.

**Additional file 19 Table S18.** Genome-wide detection of highly differentiated loci between DW and UP-XI.

**Additional file 20 Table S19.** Genome-wide detection of highly differentiated loci between IR-XI and UP-XI.

**Additional file 21 Table S20.** Genome-wide detection of highly differentiated loci between IR-GJ and UP-GJ.

**Additional file 22 Table S21.** Significant association signals for deepwater in the full populaiton detected using FaST-LMM.

**Additional file 23 Table S22.** The cloned genes for deepwater and heat map of the ratio of RPKM.

**Additional file 24 Table S23.** Haplotype analysis of the candidate genes for deepwater QTLs.

## Data Availability

All the genomic data of 867 rice accessions from 3 K RG can be downloaded from https://aws.amazon.com/public-data-sets/3000-rice-genome/. All raw DNA-Seq data sets of 140 wild and one cultivated rice accessions were downloaded from the NCBI SRA database (https://www.ncbi.nlm.nih.gov/sra), and the details of the accession numbers were included in Additional file 1: Table S1. All data generated or analyzed during this study are included in this published article or its supplementary information files or are available from the corresponding authors on reasonable request.

## Abbreviations

Or: *Oryza rufipogon*
On: *Oryza nivara*
Ob: *Oryza barthii*
Ol: *Oryza longistaminata*
Om: *Oryza meridionalis*
Oglu: *Oryza glumaepatula*
Ogla: *Oryza glaberrima*
ROD: Reduction of diversity
RAiSD: Raised Accuracy in Sweep Detection
ML: Maximum likelihood
FaST-LMM: Factored spectrally transformed linear mixed models
DW: Deepwater rice
SW: Swamp rice
TW: Tidal wetland rice
IR: Irrigated rice
RL: Rainfed lowland rice
UP: Upland rice
OrI: Indica-type wild rice
OrII: Japonica-type wild rice
PC: Principal component
IR-GJ: Irrigated *geng (japonica)* rice
RL-GJ: Rainfed lowland *geng (japonica)* rice
UP-GJ: Upland *geng (japonica)* rice
IR-XI: Irrigated *xian (indica)* rice
RL-XI: Rainfed lowland *xian (indica)* rice
SW-XI: Swamp *xian (indica)* rice
UP-XI: Upland *xian (indica)* rice
DW-XI: Deepwater *xian (indica)* rice
AFD: Alternative allele frequency differentiation
XI: *Xian (indica)* rice
GJ: *Geng (japonica)*
RPKM: Reads Per Kilobase per Million mapped reads
RPKM_Deepwater_: The RPKM of deepwater rice accessions subjected to complete submergence
RPKM_Non-deepwater_: The RPKM of non-deepwater rice accessions subjected to complete submergence
RPKM_after/before_: The ratio of RPKM in the after to before submergence of the deepwater variety.
