# Supplementary material for: Evolution of different rice ecotypes and genetic basis of flooding adaptability in Deepwater rice by GWAS
Source: BMC Plant Biol. 2022 Nov 14;22:526. doi: 10.1186/s12870-022-03924-y (PMC9661789; doi:10.1186/s12870-022-03924-y)
Supplement: Supplementary file 3 — Additional file 3 Fig. S1 PCA plot of the first two eigenvectors (a) and two and three eigenvectors (b) of rice accessions. Additional file 2. Fig. S2 Maximum-likelihood tree of rice assuming 0 to 3 migration edges. Additional file 2. Fig. S3 Corresponding residual of rice assuming 0 to 10 migration edges. Additional file 2. Fig. S4 Genome-wide detection of positive selection in different rice ecotypes. Additional file 2. Fig. S5 Selective sweep regions identified by the greatest reduction of diversity (ROD) of UP-GJ. Additional file 2. Fig. S6 Selective sweep regions identified by the greatest reduction of diversity (ROD) of IR-XI. Additional file 2. Fig. S7 Selective sweep regions identified by the greatest reduction of diversity (ROD) of IR-GJ. Additional file 2. Fig. S8 Genomic differentiation regions identified by the greatest relative divergence (FST) between IR-XI and UP-XI populations. Additional file 2. Fig. S9 Genomic differentiation regions identified by the greatest relative divergence (FST) between IR-GJ and UP-GJ populations. Additional file 2. Fig. S10 Quantile-quantile plots and Manhattan plots for the GWAS in the full populations using FaST-LMM. [file 12870_2022_3924_MOESM3_ESM.docx]

**Supplementary Information**

**Title:** Evolution of different rice ecotypes and genetic basis of flooding adaptability in deepwater rice by GWAS

Xueqiang Wang^1,3,7†^, Yan Zhao^2†^, Conghui Jiang^4†^, Libing Wang^3†^, Lei Chen^5^, Fengmei Li^3^, Yanhong Zhang^6^, Yinghua Pan^5*^ and Tianzhen Zhang^1*^

^1^ Agronomy Department, College of Agriculture and Biotechnology, Zhejiang University, Hangzhou, 310058, PR China.

^2^ State Key Laboratory of Crop Biology, Shandong Key Laboratory of Crop Biology, College of Agronomy, Shandong Agricultural University, Tai'an, Shandong, 271018, PR China.

^3^ Hainan Yazhou Bay Seed Laboratory, Sanya, Hainan, 572025, PR China.

^4^ Shandong Rice Research Institute, Shandong Academy of Agricultural Sciences, Jinan 250100, China.

^5^ Rice Research Institute, Guangxi Academy of Agricultural Sciences/Guangxi Key Laboratory of Rice Genetics and Breeding, Nanning, 530007, Guangxi, China.

^6^ Institute of Nuclear and Biological Technologies, Xinjiang Academy of Agricultural Sciences, Urumqi, 830091, China.

^7^ Hainan Institute of Zhejiang University, Sanya, Hainan, 572025, PR China.

^†^ These authors have contributed equally to this work.

^*^ Correspondence should be addressed to Yinghua Pan (Email: panyinghua2008@163.com) and Tianzhen Zhang (Email: cotton@zju.edu.cn).

**Additional file 1. Fig. S1** PCA plot of the first two eigenvectors (**a**) and two and three eigenvectors (**b**) of rice accessions.

**Additional file 1. Fig. S2** Maximum-likelihood tree of rice assuming 0 to 3 migration edges.

**Additional file 1. Fig. S3** Corresponding residual of rice assuming 0 to 10 migration edges.

**Additional file 1. Fig. S4** Genome-wide detection of positive selection in different rice ecotypes.

**Additional file 1. Fig. S5** Selective sweep regions identified by the greatest reduction of diversity (ROD) of UP-GJ.

**Additional file 1. Fig. S6** Selective sweep regions identified by the greatest reduction of diversity (ROD) of IR-XI.

**Additional file 1. Fig. S7** Selective sweep regions identified by the greatest reduction of diversity (ROD) of IR-GJ.

**Additional file 1. Fig. S8** Genomic differentiation regions identified by the greatest relative divergence (*F_ST_*) between IR-XI and UP-XI populations.

**Additional file 1. Fig. S9** Genomic differentiation regions identified by the greatest relative divergence (*F_ST_*) between IR-GJ and UP-GJ populations.

**Additional file 1. Fig. S10** Quantile-quantile plots and Manhattan plots for the GWAS in the full populations using FaST-LMM.

**Additional file 2. Table S1.** Information about samples used in this study.

**Additional file 3. Table S2.** Summary of Annotation for genomic variation.

**Additional file 4. Table S3.** Genetic divergence (*F_ST_*) among distinct rice ecotypes populations and wild rice.

**Additional file 5. Table S4.** The mean (θπ) of different subpopulations in cultivated and wild rice.

**Additional file 6. Table S5.** Variation types between each ecotype and its immediate ancestral progenitor.

**Additional file 7. Table S6.** Genome-wide detection of positive selection in deepwater (DW).

**Additional file 8. Table S7.** Genome-wide detection of positive selection in upland XI rice.

**Additional file 9. Table S8.** Genome-wide detection of positive selection in upland GJ rice.

**Additional file 10. Table S9.** Genome-wide detection and functional annotation of selective sweep regions in the deepwater rice (DW).

**Additional file 11. Table S10.** Genome-wide detection and functional annotation of selective sweep regions in the upland XI rice.

**Additional file 12. Table S11.** Genome-wide detection and functional annotation of selective sweep regions in the upland GJ rice.

**Additional file 13. Table S12.** KEGG annotation of selective sweep regions in the deepwater rice (DW).

**Additional file 14. Table S13.** KEGG annotation of selective sweep regions in the upland XI rice.

**Additional file 15. Table S14.** KEGG annotation of selective sweep regions in the upland GJ rice.

**Additional file 16. Table S15.** Genome-wide detection and functional annotation of selective sweep regions in the irrigated XI rice.

**Additional file 17. Table S16.** Genome-wide detection and functional annotation of selective sweep regions in the irrigated GJ rice.

**Additional file 18. Table S17.** Ratio of RPKM within the 200-kb selective sweep region on chromosome 11.

**Additional file 19. Table S18.** Genome-wide detection of highly differentiated loci between DW and UP-XI.

**Additional file 20. Table S19.** Genome-wide detection of highly differentiated loci between IR-XI and UP-XI.

**Additional file 21. Table S20.** Genome-wide detection of highly differentiated loci between IR-GJ and UP-GJ.

**Additional file 22. Table S21.** Significant association signals for deepwater in the full populaiton detected using FaST-LMM.

**Additional file 23. Table S22.** The cloned genes for deepwater and heat map of the ratio of RPKM.

**Additional file 24. Table S23.** Haplotype analysis of the candidate genes for deepwater QTLs.


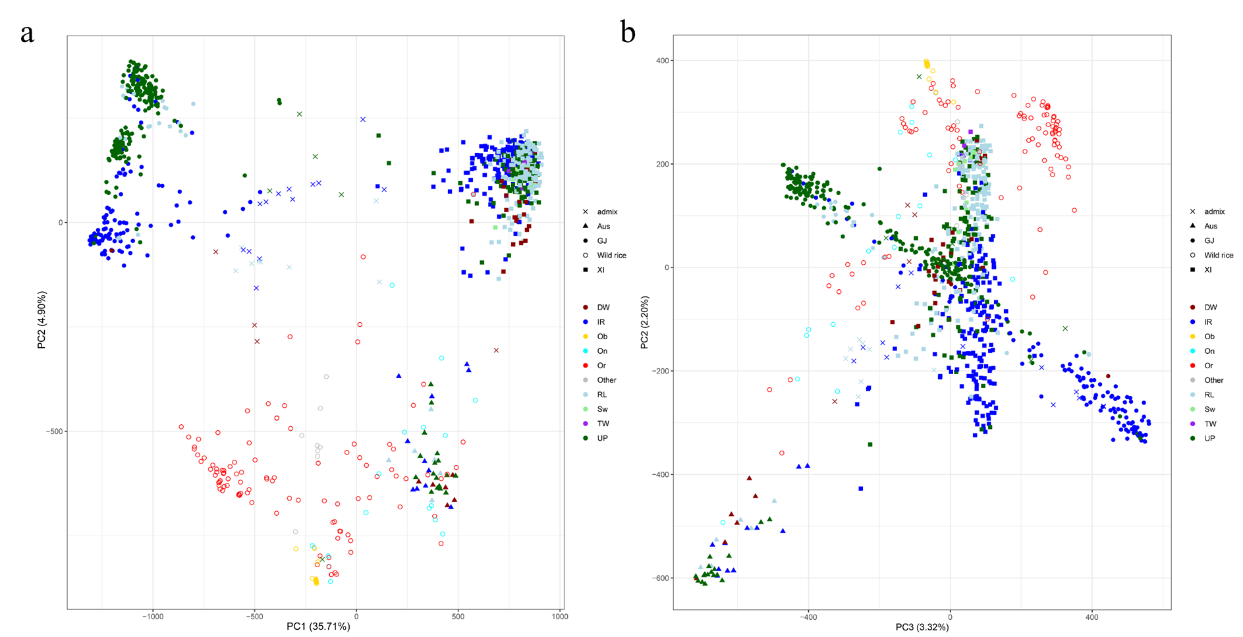


**Fig. S1** PCA plot of the first two eigenvectors (**a**) and two and three eigenvectors (**b**) of rice accessions. The dots color of abbreviations for the major rice subgroups are the same as in **Fig. 1**.


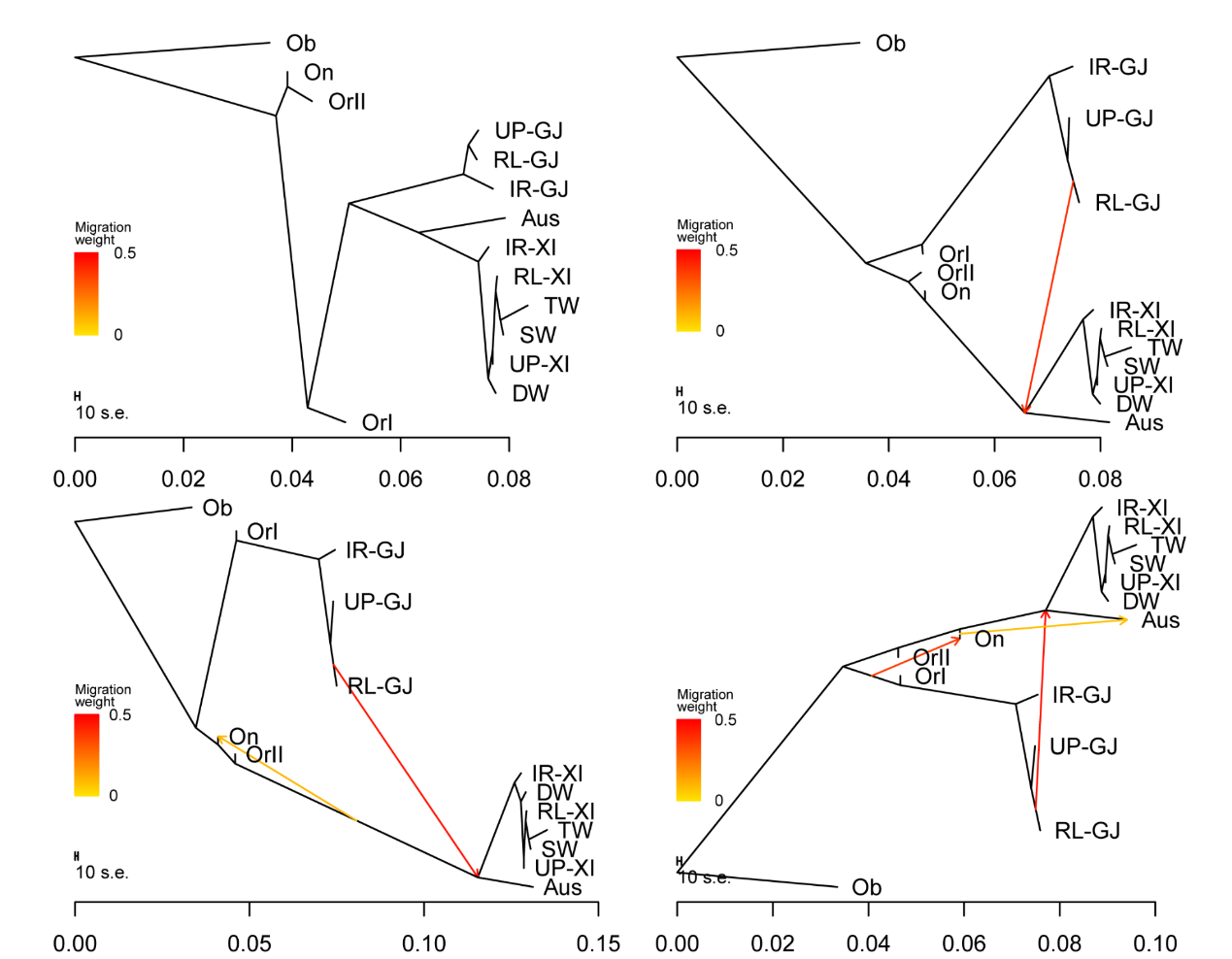


**Fig. S2** Maximum-likelihood tree of rice assuming 0 to 3 migration edges. The wild rice population was divided into four subpopulations (*Oryza rufipogon* (OrI and OrII), *Oryza nivara* (On) and *Oryza barthii* (Ob)). The abbreviations for the major domesticated rice subgroups are same as **Fig. 2**. The bootstrap values on the tree are based on 10000 replicates. Arrows on the graph represent admixture events among different rice populations. The scale bar shows ten times the average standard error of the entries in the sample covariance matrix.


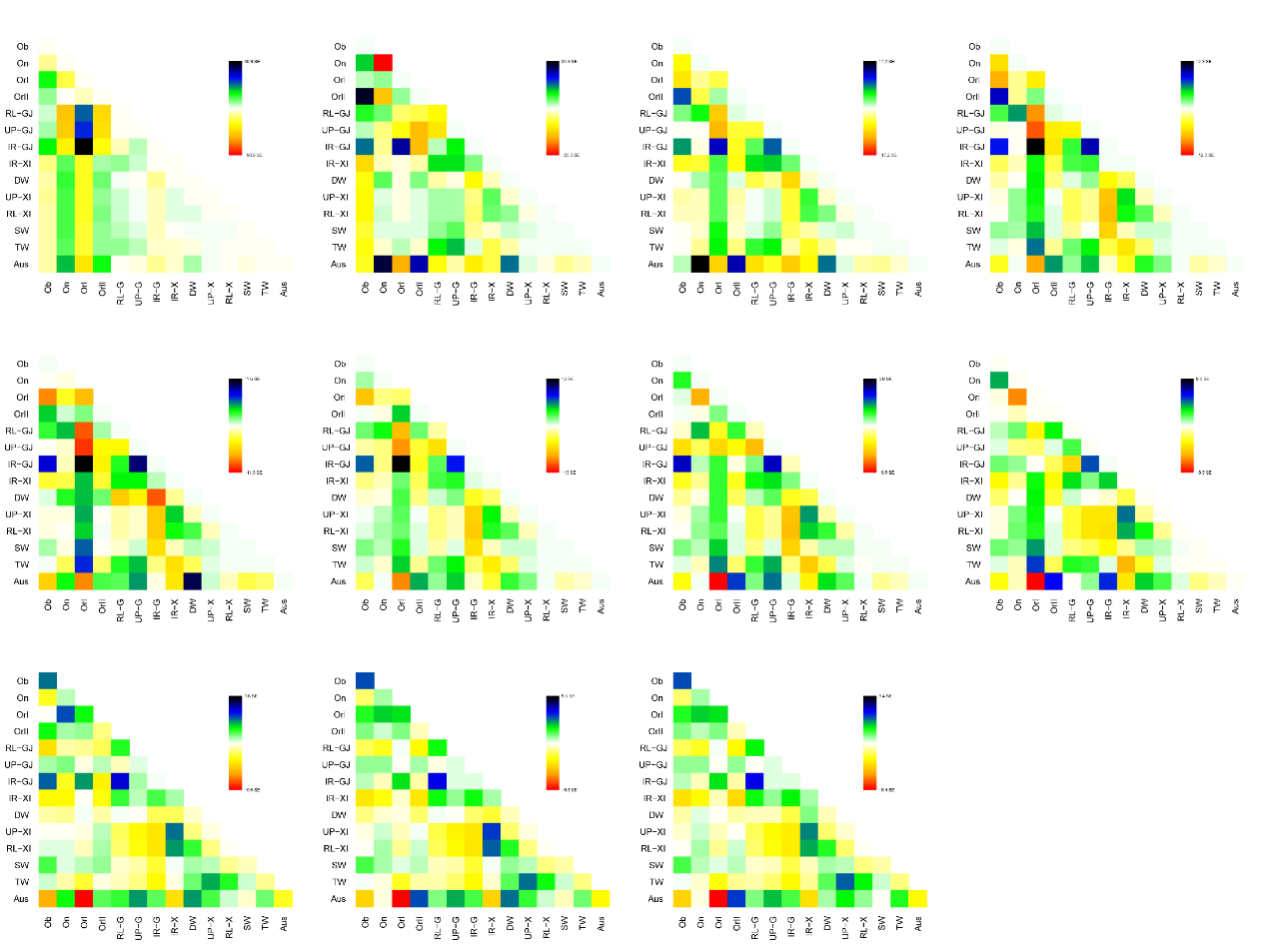


**Fig. S3** Corresponding residual of rice assuming 0 to 10 migration edges. The abbreviations for the major domesticated rice subgroups are same as **Fig. 2**.


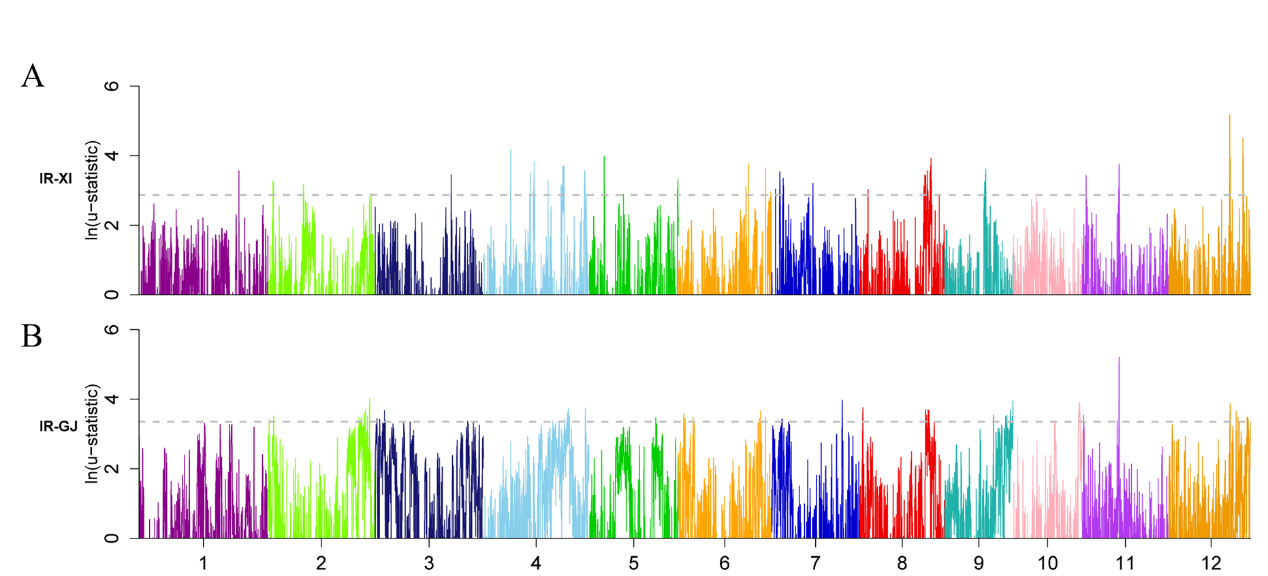


**Fig. S4** Genome-wide detection of positive selection in different rice ecotypes. **A** irrigated *indica* rice (IR-XI) **B** irrigated *japonica* rice (IR-GJ). Dashed lines indicate the top 1% of the μ-statistic values.


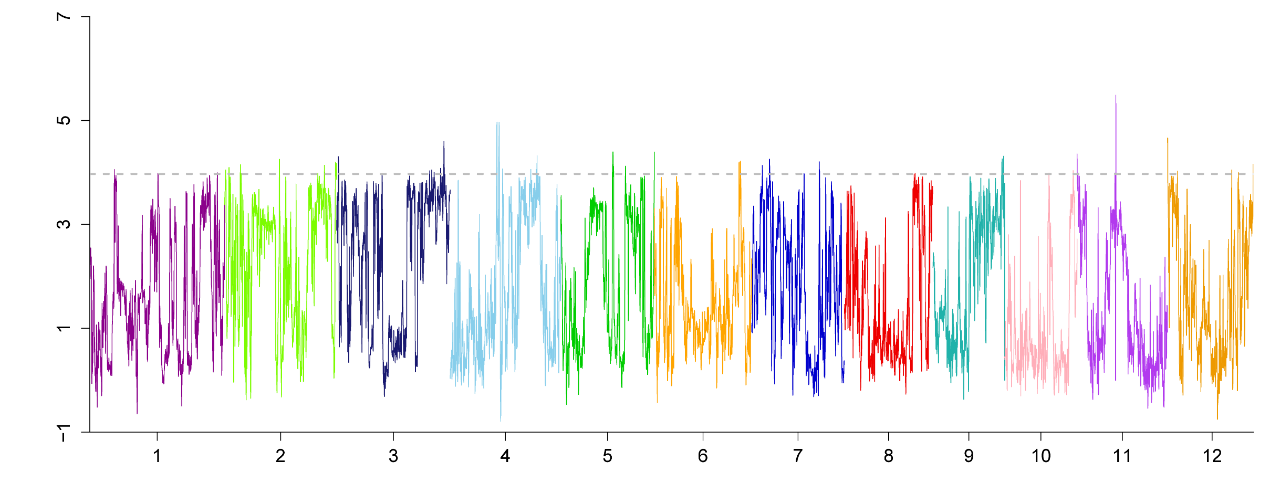


**Fig. S5** Selective sweep regions identified by the greatest reduction of diversity (ROD) of UP-GJ. Dashed lines indicate the top 1% of the ROD values.


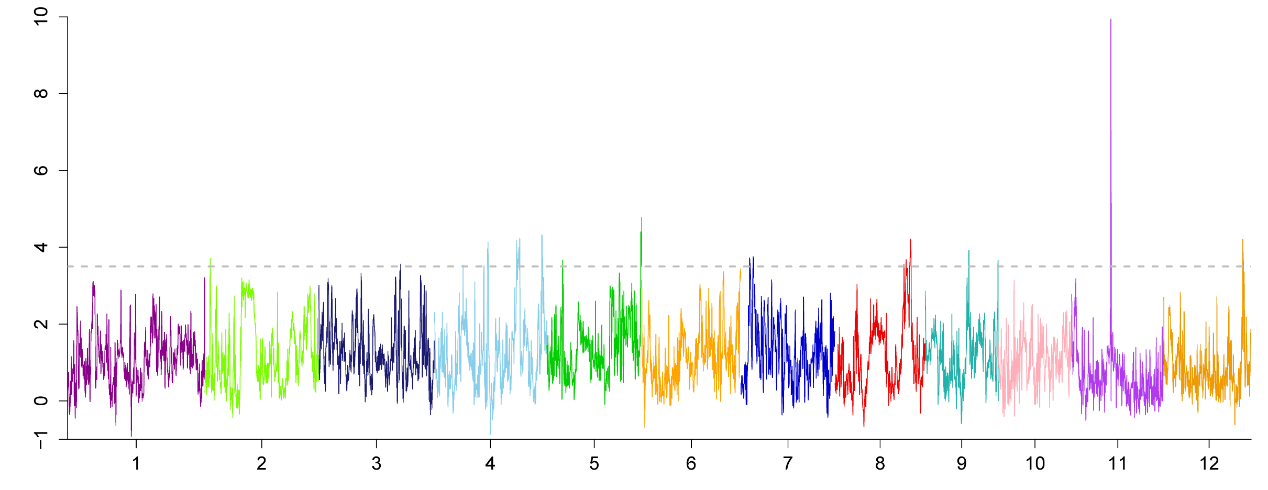


**Fig. S6** Selective sweep regions identified by the greatest reduction of diversity (ROD) of IR-XI. Dashed lines indicate the top 1% of the ROD values.


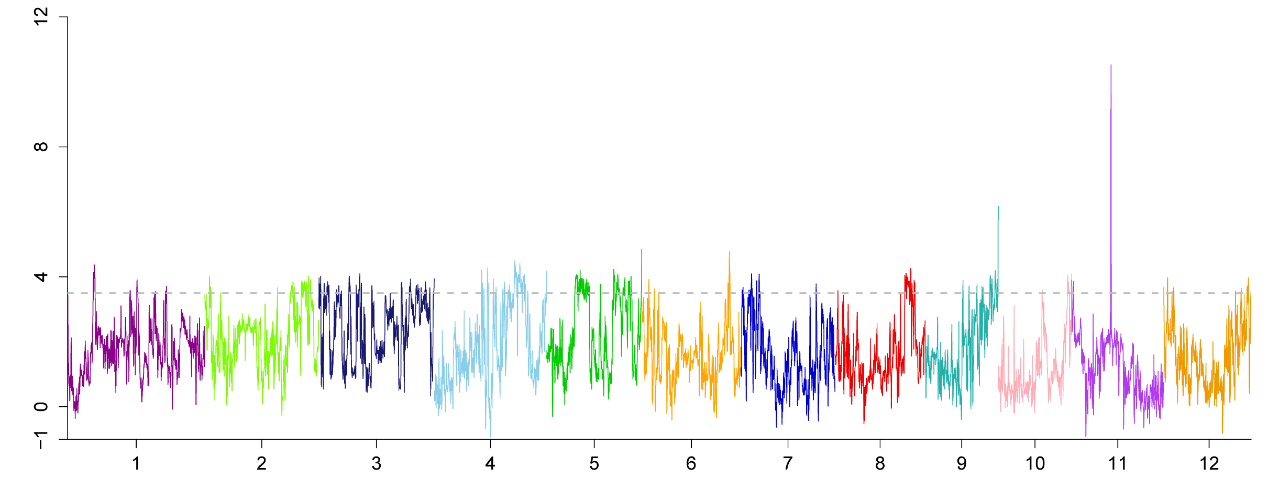


**Fig. S7** Selective sweep regions identified by the greatest reduction of diversity (ROD) of IR-GJ. Dashed lines indicate the top 1% of the ROD values.


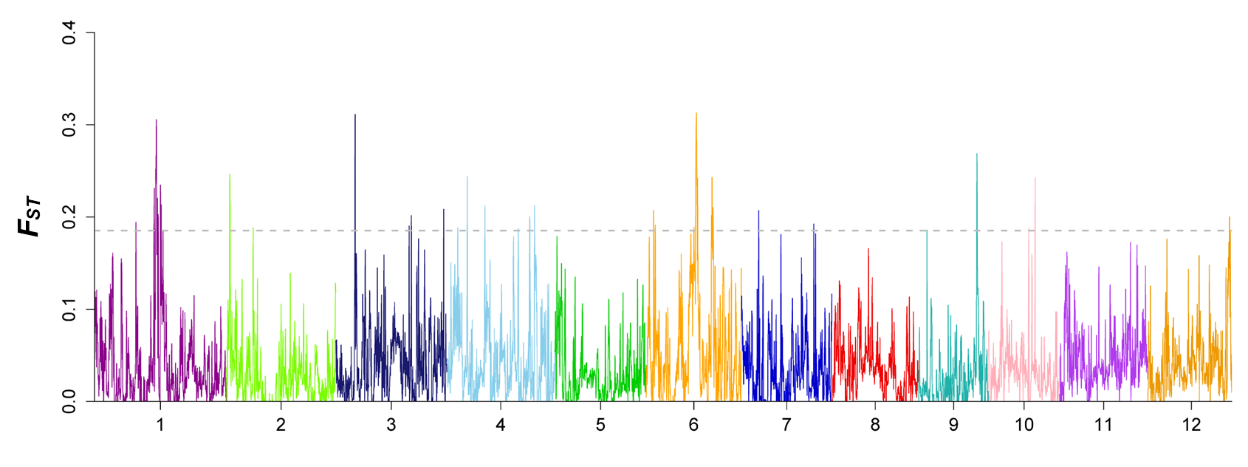


**Fig. S8** Genomic differentiation regions identified by the greatest relative divergence (*F_ST_*) between IR-XI and UP-XI populations. Dashed lines indicate the top 1% of the *F_ST_* values.


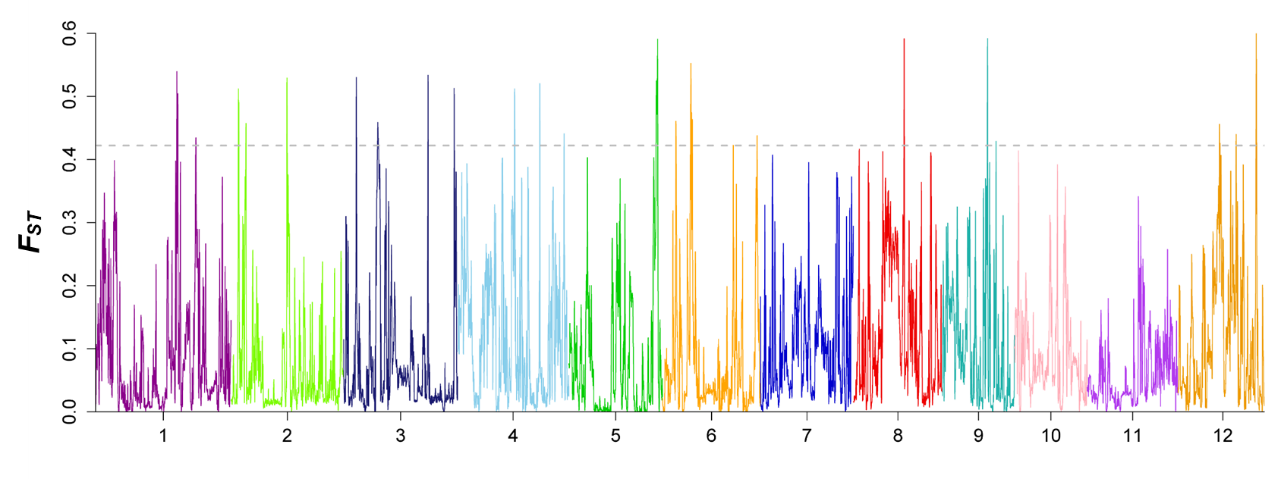


**Fig. S9** Genomic differentiation regions identified by the greatest relative divergence (*F_ST_*) between IR-GJ and UP-GJ populations. Dashed lines indicate the top 1% of the *F_ST_* values.


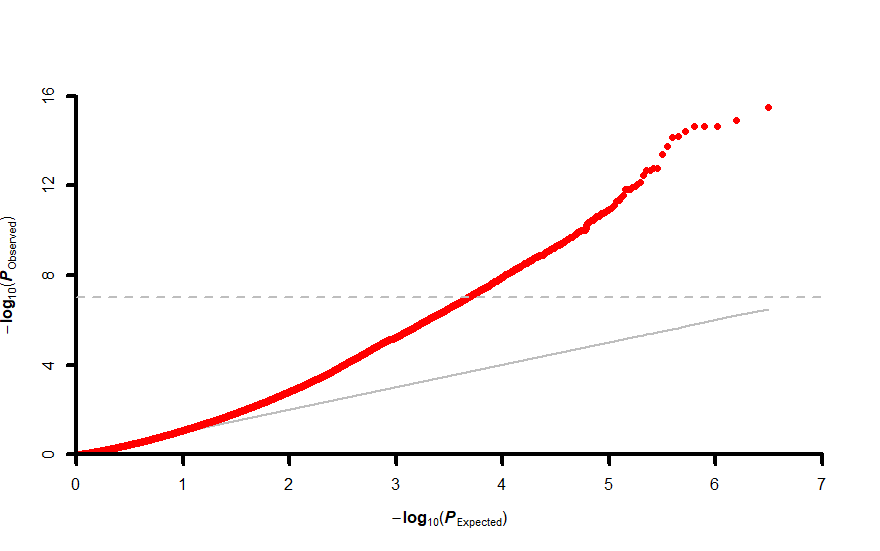


**Fig. S10** Quantile-quantile plots and Manhattan plots for the GWAS in the full populations using FaST-LMM.
